# Catastrophic Hypoxic-Ischemic Brain Injury Following Influenza A Illness in a Young Woman: A Case Report

**DOI:** 10.7759/cureus.107743

**Published:** 2026-04-26

**Authors:** Cameron Bondy, Sharon Wang, Sarkis Arabian

**Affiliations:** 1 Internal Medicine, Arrowhead Regional Medical Center, Colton, USA; 2 Infectious Disease, Arrowhead Regional Medical Center, Colton, USA; 3 Critical Care, Arrowhead Regional Medical Center, Colton, USA

**Keywords:** acute respiratory failure, anoxic brain injury, cerebellar tonsillar herniation, diffuse cerebral edema, hypoxic–ischemic brain injury, intracranial hypertension, sepsis, viral prodrome

## Abstract

Seasonal viral respiratory infections such as influenza are typically self-limited but can, in rare instances, precipitate severe systemic and neurologic complications. We report the case of a previously healthy 27-year-old woman who developed rapid neurologic collapse one week after flu symptoms and testing positive for influenza A. She presented with altered mental status and experienced a witnessed seizure requiring emergent intubation, followed by refractory shock necessitating multiple vasopressors. Neuroimaging demonstrated diffuse cerebral edema with cerebellar tonsillar herniation and hyperdensity of the basal cisterns, with subsequent computerized tomography (CT) angiography revealing absence of cerebral perfusion. Neurologic examination remained consistent with profound brain injury, including a Glasgow Coma Scale score of 3, consisting of no eye opening, no motor response, and no verbal response. Her clinical course was consistent with hypoxic-ischemic brain injury secondary to systemic hypoperfusion and hypoxemia in the setting of viral illness and septic shock. The patient ultimately died following withdrawal of life-sustaining therapy. This case highlights the potential for viral prodromes to trigger catastrophic neurologic injury through systemic inflammatory, hypoxic, and hemodynamic mechanisms, even in young patients without preexisting disease. Early recognition of malignant cerebral edema, pseudo-subarachnoid hemorrhage, and absent cerebral perfusion on advanced imaging is critical for prognostication and timely goals-of-care discussions. Additionally, the patient had positive cocaine test results, raising the possibility that sympathomimetic toxicity or cocaine-associated vasculopathy may have contributed to the severity of cerebral edema. Finally, this case underscores the importance of influenza vaccination as a potentially preventable factor in rare but devastating neurologic complications of viral illness.

## Introduction

Seasonal viral respiratory infections, including influenza, are typically self-limited illnesses but may occasionally precipitate severe systemic and neurologic complications. Influenza and other viral syndromes have been associated with sepsis, acute respiratory failure, cytokine-mediated endothelial injury, and profound hypoxemia, all of which can contribute to secondary central nervous system injury [1-3]. In rare cases, these processes culminate in diffuse cerebral edema, critically elevated intracranial pressure, and fatal brain herniation, even in young patients without underlying medical comorbidities [4].

Hypoxic-ischemic brain injury (HIBI) represents a final common pathway of global cerebral insult resulting from systemic hypoperfusion, hypoxemia, or cardiopulmonary failure [5]. When severe, HIBI may rapidly progress to malignant cerebral edema, loss of cerebral autoregulation, and cessation of cerebral blood flow [5,6]. Early recognition of this trajectory is challenging, particularly when neurologic deterioration occurs in the context of a presumed benign viral illness [4]. In addition, stimulant use, particularly cocaine, has been independently associated with acute neurologic injury through mechanisms including cerebral vasoconstriction, impaired autoregulation, endothelial dysfunction, and increased risk of ischemia, hemorrhage, and seizure activity, which may further compound global cerebral injury [5].

We present the case of a previously healthy 27-year-old woman who developed rapid neurologic collapse following a diagnosis of influenza A, progressing to seizure, shock, diffuse cerebral edema, and cerebellar tonsillar herniation with absent cerebral perfusion. Notably, her only significant history was cocaine use, which may have exacerbated the severity of her clinical course.

## Case presentation

A 27-year-old woman with no known past medical history was transferred from an outside hospital for higher-level care after neuroimaging demonstrated diffuse cerebral edema with cerebellar tonsillar herniation and hyperdensity within the basal cisterns. Review of the outside hospital records revealed a positive influenza A polymerase chain reaction (PCR) approximately one week prior to presentation. She subsequently presented with altered mental status, cough, nausea, and vomiting. While in the emergency department, the patient experienced a witnessed seizure and was emergently intubated for airway protection. 

Initial laboratory studies from the outside hospital are shown in Table 1. Noteworthy findings included marked leukocytosis with neutrophilic predominance, significantly elevated lactate, and a high anion gap metabolic acidosis. Additional abnormalities included hyperglycemia and an elevated D-dimer level, while renal function remained preserved. Toxicology screening was positive for cocaine and negative for other tested substances, including amphetamines, barbiturates, tetrahydrocannabinol (THC), and benzodiazepines. Additional testing, including blood cultures, SARS-CoV-2 testing, procalcitonin, and a comprehensive respiratory viral panel, was negative.

**Table 1:** Initial laboratory tests eGFR: estimated glomerular filtration rate; BUN: blood urea nitrogen; THC: tetrahydrocannabinol; MCHC: mean corpuscular hemoglobin concentration; MCH: mean corpuscular hemoglobin; RBC: red blood cell; WBC: white blood cell

Test	Patient Value	Normal Range
Lactate	7.6 mmol/L	0.5–2.0 mmol/L
WBC count	22.3 ×10³/µL	4.0–11.0 ×10³/µL
Neutrophils (%)	91.30%	40–70%
Lymphocytes (%)	5.40%	10–50%
Monocytes (%)	3.10%	0–12%
Eosinophils (%)	0.00%	0–7%
Basophils (%)	0.20%	0–2%
Neutrophils (Absolute)	20.3 ×10³/µL	1.6–8.6 ×10³/µL
Lymphocytes (Absolute)	1.2 ×10³/µL	0.4–5.4 ×10³/µL
Monocytes (Absolute)	0.7 ×10³/µL	0–1.3 ×10³/µL
Eosinophils (Absolute)	0.0 ×10³/µL	0–0.8 ×10³/µL
Basophils (Absolute)	0.0 ×10³/µL	0–0.2 ×10³/µL
Nucleated RBCs	0.10%	0%
RBC count	5.37 ×10⁶/µL	4.0–5.2 ×10⁶/µL
Hemoglobin	14.6 g/dL	12.2–16.2 g/dL
Hematocrit	46.30%	36–46%
Mean corpuscular volume	86.2 fL	80–100 fL
MCH	27.2 pg	28–32 pg
MCHC	31.5 g/dL	32–36 g/dL
Red cell distribution width	14.90%	11.8–14.3%
Platelet count	273 ×10³/µL	140–450 ×10³/µL
Mean platelet volume	9.3 fL	6.9–10.8 fL
Sodium	142 mmol/L	136–145 mmol/L
Potassium	3.9 mmol/L	3.5–5.1 mmol/L
Chloride	106 mmol/L	98–107 mmol/L
HCO_3_^−^ (Bicarbonate)	16 mmol/L	22–28 mmol/L
Anion Gap	20	8–12
BUN	8 mg/dL	9–23 mg/dL
Creatinine	0.66 mg/dL	0.55–1.02 mg/dL
eGFR	123 mL/min	>90 mL/min
BUN/Creatinine ratio	12.1	10–20
Glucose	194 mg/dL	70–140 mg/dL (non-fasting)
Calcium	9.4 mg/dL	8.7–10.4 mg/dL
D-dimer	1.23 mg/L FEU	<0.50 mg/L FEU
Toxicology – Cocaine	Positive	Negative
Toxicology – Amphetamines	Negative	Negative
Toxicology – Barbiturates	Negative	Negative
Toxicology – THC	Negative	Negative
Toxicology – Benzodiazepines	Negative	Negative

**Figure 1:**
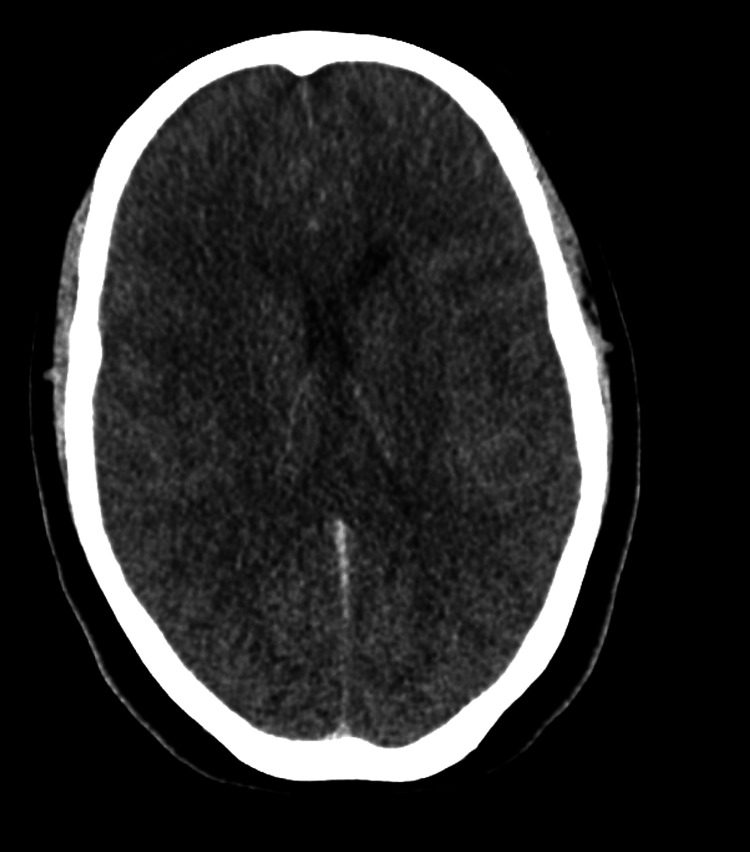
CT head superior slice Diffuse cerebral edema with sulcal effacement and cerebral/cerebellar swelling is observed, resulting in effacement of the lateral (posterior and temporal horns) and fourth ventricles, along with downward displacement of the cerebellar tonsils. There is increased density within the basilar cisterns and along the tentorium.

**Figure 2:**
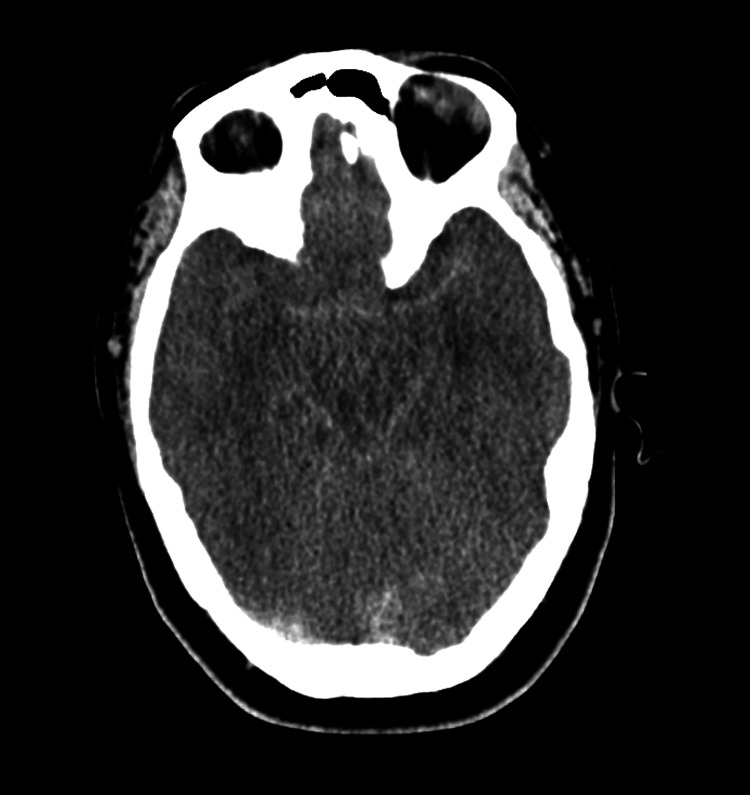
CT head orbital slice Diffuse cerebral edema with loss of gray–white differentiation and sulcal effacement is observed, along with effacement of the basal cisterns, consistent with increased intracranial pressure.

**Figure 3:**
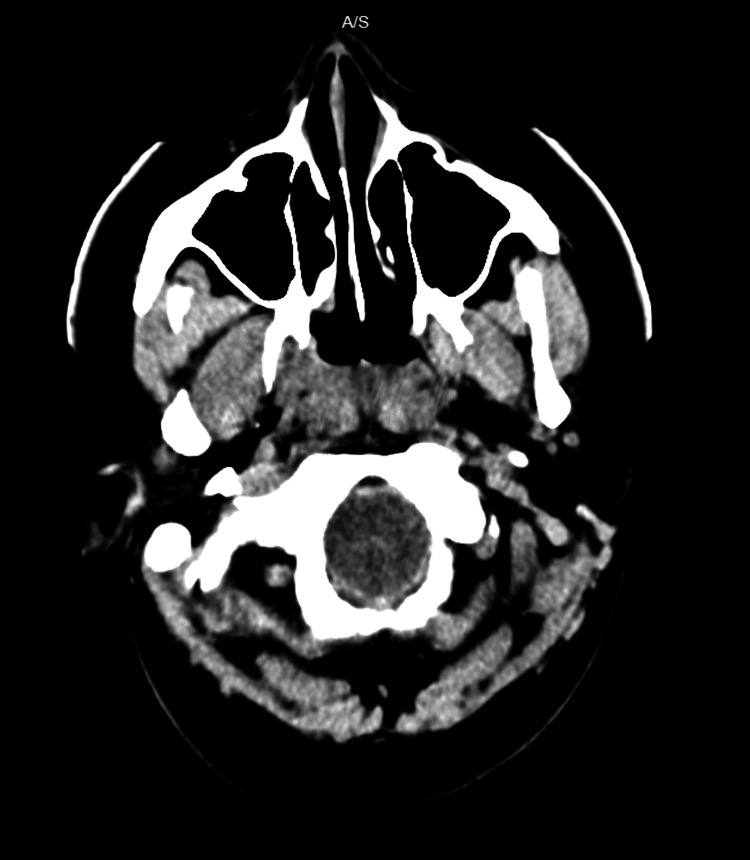
CT head inferior slice Crowding at the skull base is observed with effacement of the basal cisterns and apparent compression at the level of the foramen magnum, concerning for downward herniation.

**Figure 4:**
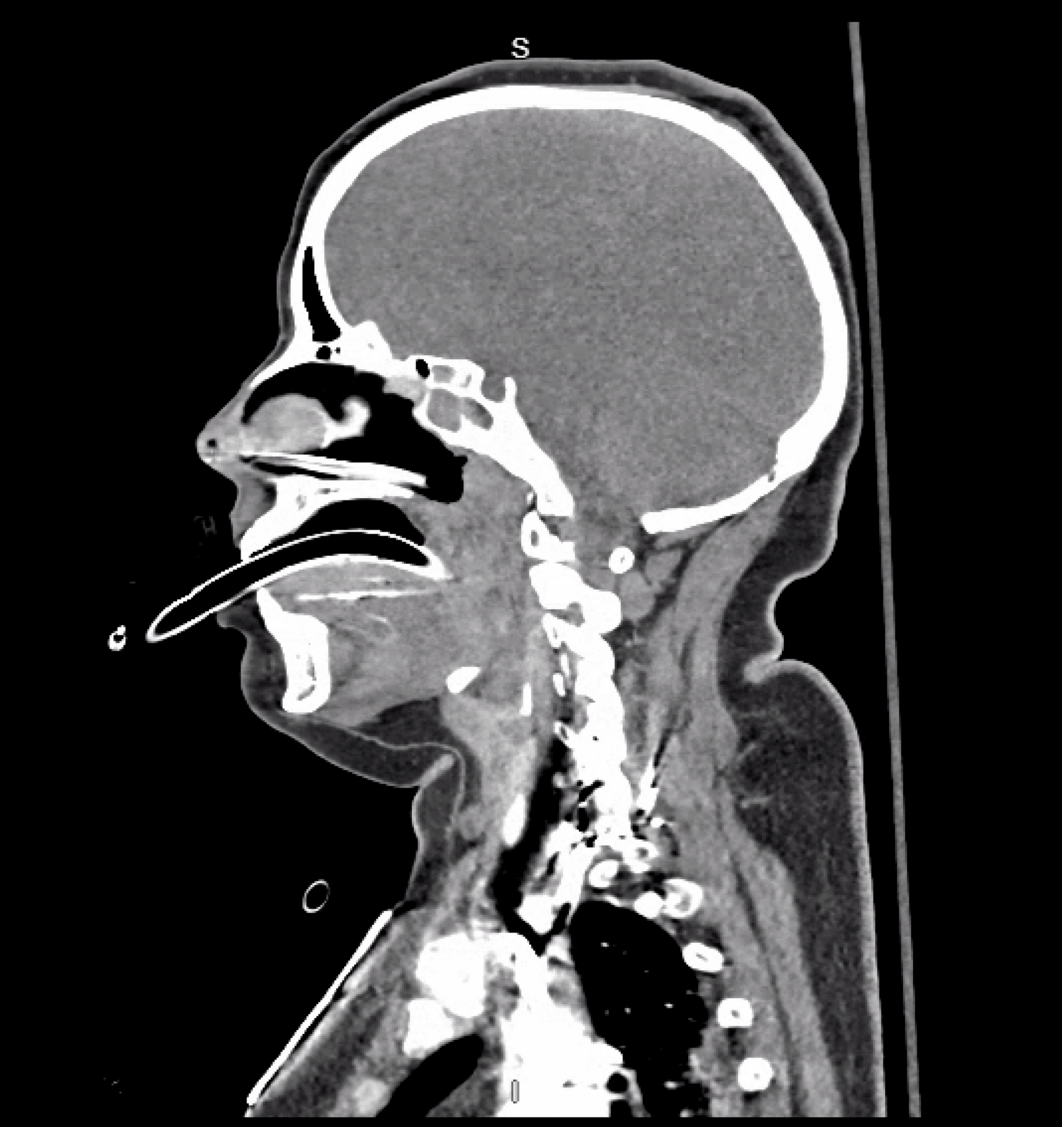
CT angiogram head and neck (sagital view) Preserved opacification of the extracranial carotid and vertebral arterial systems is observed, with absence of intracranial arterial filling. There is no visualization of the intracranial internal carotid arteries, circle of Willis, or distal cerebral branches. These findings are consistent with absent cerebral perfusion in the setting of markedly elevated intracranial pressure.

Non-contrast computerized tomography (CT) of the head (Figures 1-3) demonstrated diffuse cerebral edema, sulcal and ventricular effacement, and downward cerebellar tonsillar herniation with hyperdensity of the basal cisterns. CT angiography of the head and neck demonstrated the absence of cerebral perfusion (Figure 4). 

CT chest (Figure 4) demonstrated extensive bilateral airspace disease, predominantly involving the dependent posterior lower lobes. There were multifocal ground-glass opacities with superimposed areas of consolidation, producing a mixed alveolar-interstitial pattern. The abnormalities were symmetric and posterior-predominant, with relative sparing of the anterior lung fields on this slice. No discrete pulmonary nodules, cavitation, pneumothorax, or large pleural effusions were identified. The central airways appeared patent, and there was no obvious mediastinal abnormality at this level.

**Figure 5:**
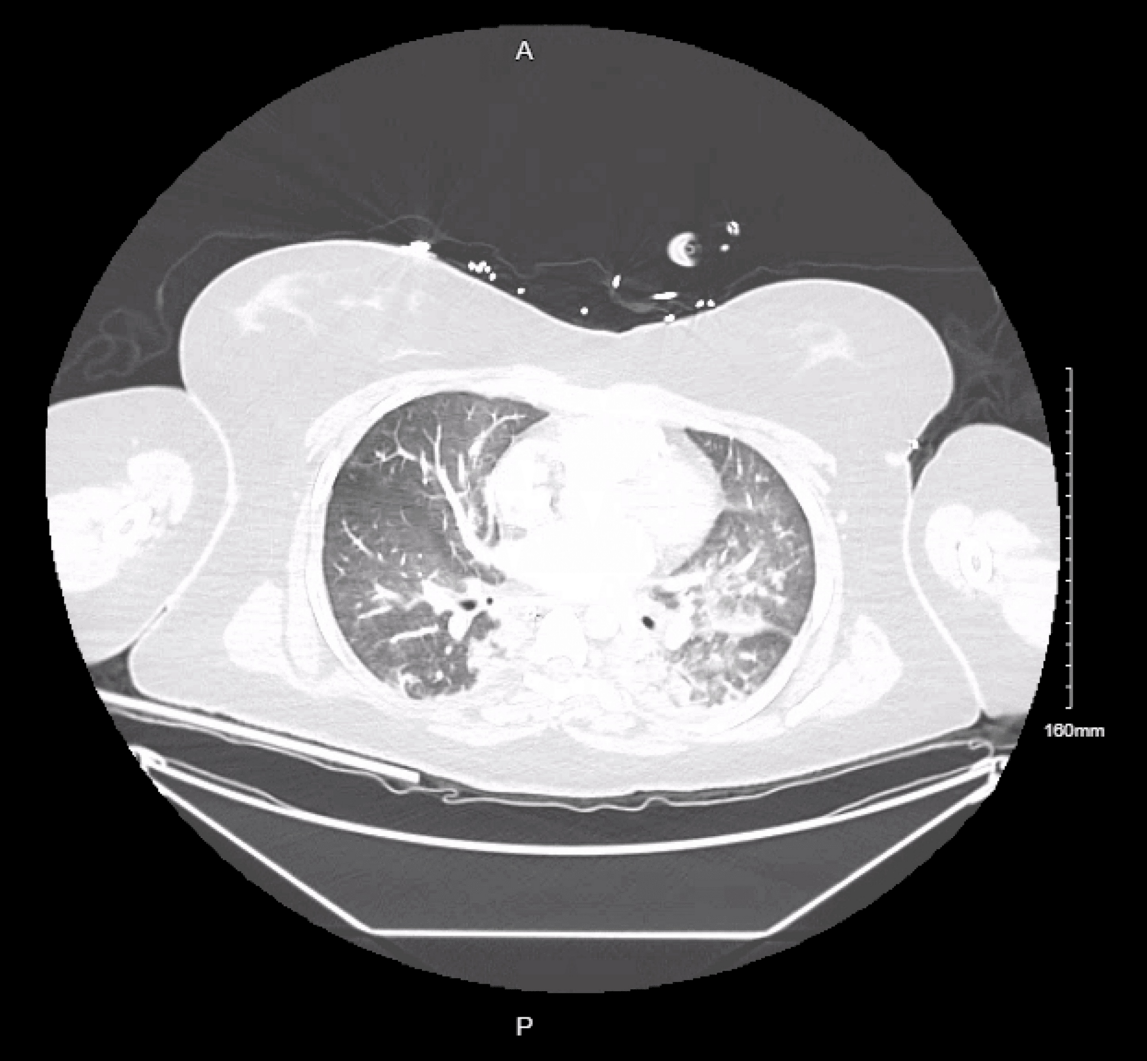
CT chest (axial view) Bilateral, posterior-predominant airspace disease is seen with multifocal ground-glass opacities and superimposed consolidation, consistent with a mixed alveolar–interstitial pattern. Findings are symmetric with relative anterior sparing. No nodules, cavitation, pneumothorax, or large pleural effusions are identified, and the central airways are patent.

The patient was received at our hospital mechanically ventilated in pressure-control assist-control mode (respiratory rate 14 breaths per minute, inspiratory pressure 20 cm H₂O, positive end-expiratory pressure 8 cm H₂O, fraction of inspired oxygen 100%). Neurologic examination revealed a Glasgow Coma Scale (GCS) score of 3T with fixed, dilated, and nonreactive pupils. On arrival, she was hypotensive and tachycardic, requiring initiation of vasopressor support. She was admitted to the medical intensive care unit. 

The patient remained mechanically ventilated and required escalating vasopressor support, including norepinephrine, phenylephrine, and epinephrine. Neurosurgical consultation documented the absence of brainstem reflexes, and ultimately, no surgical intervention was recommended. Sedation was discontinued without a change in serial neurologic examinations. She remained a persistent GCS 3T with absent brainstem reflexes. Following discussions with the patient’s family, her code status was changed to do-not-resuscitate (DNR). On hospital day two, the patient developed worsening hypotension with increasing vasopressor requirements and subsequently lost pulses. She was pronounced deceased.

## Discussion

This report described the case of a previously healthy young woman who rapidly progressed from a viral prodrome to seizure, refractory shock, and catastrophic neurologic injury. Her presentation was notable for severe lactic acidosis, neutrophilic leukocytosis, vasopressor-dependent hypotension, and acute hypoxemic respiratory failure with extensive bilateral pulmonary opacities, consistent with septic shock and severe respiratory compromise [2,3]. CT of the chest demonstrated bilateral, posterior-predominant ground-glass opacities and consolidations, a pattern concerning for a severe infectious process such as multifocal pneumonia. Given the dependent distribution and the patient’s altered mental status and subsequent intubation, aspiration pneumonia or aspiration pneumonitis represents an additional consideration, while viral pneumonia remains plausible.

Neuroimaging findings were most consistent with severe HIBI complicated by diffuse cerebral edema and transtentorial and tonsillar herniation [6,7]. Initial non-contrast CT raised concern for subarachnoid hemorrhage due to hyperdensity within the basal cisterns; however, serial imaging demonstrated progressive diffuse sulcal and ventricular effacement with worsening cerebral and cerebellar edema. In the context of malignant cerebral edema, basal cisternal hyperdensity is a recognized manifestation of pseudo-subarachnoid hemorrhage, a radiographic mimic resulting from venous congestion and reduced parenchymal attenuation rather than true hemorrhage [8,9]. Recognition of this entity is critical, as it shifts diagnostic focus away from aneurysmal hemorrhage toward global anoxic injury.

CT angiography further clarified the severity and irreversibility of neurologic injury by demonstrating the absence of intracranial arterial opacification, consistent with cessation of cerebral blood flow due to critically elevated intracranial pressure rather than primary large-vessel occlusion [10]. In patients with diffuse cerebral edema, absent cerebral perfusion on CT angiography correlates with devastating neurologic injury and an extremely poor prognosis [10,11]. These imaging findings, in conjunction with the persistent absence of brainstem reflexes after cessation of sedation, supported the conclusion that neurosurgical intervention would be non-beneficial.

Alternative diagnoses were considered. Primary intracranial hemorrhage became less likely as imaging evolved toward diffuse cerebral edema with pseudo-subarachnoid appearance. Fulminant infectious meningoencephalitis was also considered; however, the presence of profound shock physiology, severe lactic acidosis, and extensive bilateral pulmonary disease favored systemic cardiopulmonary collapse with secondary HIBI as the primary pathophysiologic process [2,6]. Although urine toxicology was positive for cocaine, stimulant exposure alone does not adequately explain the combination of refractory shock, severe bilateral pneumonia, and imaging-confirmed absence of cerebral perfusion.

Overall, this case highlights the potential for viral prodromes to trigger catastrophic neurologic injury through systemic inflammatory, hypoxic, and hemodynamic mechanisms, even in young patients without preexisting disease. Early recognition of malignant cerebral edema, pseudo-subarachnoid hemorrhage, and absent cerebral perfusion on advanced imaging is critical for prognostication and timely goals-of-care discussions. In this patient, recent cocaine exposure may have contributed to the severity of cerebral injury. Cocaine is known to cause intense cerebral vasoconstriction, endothelial dysfunction, and disruption of the blood-brain barrier, mechanisms that may promote ischemia and increase vascular permeability, thereby predisposing to vasogenic cerebral edema and amplifying the effects of systemic hypoxia and inflammation [12]. Additionally, neurologic complications of influenza, including encephalitis and severe encephalopathy, have been reported in hospitalized patients with laboratory-confirmed infection, highlighting the capacity of influenza to produce direct or immune-mediated central nervous system injury [13]. In this context, preventive measures such as seasonal influenza vaccination may represent an important opportunity to reduce the risk of rare but devastating neurologic complications.

## Conclusions

This case illustrates the devastating neurologic consequences that may arise from severe viral illness, including influenza-like syndromes, even in young and previously healthy individuals. A seemingly uncomplicated viral prodrome progressed rapidly to systemic shock, hypoxia, and HIBI with malignant cerebral edema and fatal cerebellar tonsillar herniation. The absence of cerebral perfusion on advanced neuroimaging and persistent loss of brainstem reflexes confirmed irreversible global neurologic injury despite aggressive critical care. In this patient, recent cocaine exposure may have represented an additional contributing factor, as cocaine-associated vasoconstriction, endothelial injury, and disruption of the blood-brain barrier may exacerbate cerebral ischemia and increase susceptibility to cerebral edema.

Clinicians should remain alert to the potential for fulminant neurologic deterioration in patients presenting with altered mental status or seizures following viral respiratory infections. Early recognition of evolving cerebral edema and intracranial hypertension in the context of sepsis and hypoxia is essential for appropriate prognostication and timely goals-of-care discussions. This case also highlights the importance of preventive strategies, including seasonal influenza vaccination, which may reduce the risk of severe influenza infection and its rare but catastrophic neurologic complications. Viral illnesses such as influenza are not invariably benign and, in uncommon circumstances, may precipitate catastrophic brain injury through systemic inflammatory, hypoxic, and hemodynamic mechanisms.
